# Rethinking the role of the rTPJ in attention and social cognition in light of the opposing domains hypothesis: findings from an ALE-based meta-analysis and resting-state functional connectivity

**DOI:** 10.3389/fnhum.2013.00323

**Published:** 2013-07-10

**Authors:** Benjamin Kubit, Anthony I. Jack

**Affiliations:** ^1^Department of Psychology, University of CaliforniaDavis, Davis, CA, USA; ^2^Brain, Mind, and Consciousness Lab, Department of Cognitive Science, Case Western Reserve UniversityCleveland, OH, USA

**Keywords:** right temporo-parietal junction (rTPJ), attention, social cognition, opposing domains hypothesis, anti-correlations, default mode network, task positive network (TPN), functional imaging methodology

## Abstract

The right temporo-parietal junction (rTPJ) has been associated with two apparently disparate functional roles: in attention and in social cognition. According to one account, the rTPJ initiates a “circuit-breaking” signal that interrupts ongoing attentional processes, effectively reorienting attention. It is argued this primary function of the rTPJ has been extended beyond attention, through a process of evolutionarily cooption, to play a role in social cognition. We propose an alternative account, according to which the capacity for social cognition depends on a network which is both distinct from and in tension with brain areas involved in focused attention and target detection: the default mode network (DMN). Theory characterizing the rTPJ based on the area's purported role in reorienting may be falsely guided by the co-occurrence of two distinct effects in contiguous regions: activation of the supramarginal gyrus (SMG), associated with its functional role in target detection; and the transient release, during spatial reorienting, of suppression of the angular gyrus (AG) associated with focused attention. Findings based on meta-analysis and resting functional connectivity are presented which support this alternative account. We find distinct regions, possessing anti-correlated patterns of resting connectivity, associated with social reasoning (AG) and target detection (SMG) at the rTPJ. The locus for reorienting was spatially intermediate between the AG and SMG and showed a pattern of connectivity with similarities to social reasoning and target detection seeds. These findings highlight a general methodological concern for brain imaging. Given evidence that certain tasks not only activate some areas but also suppress activity in other areas, it is suggested that researchers need to distinguish two distinct putative mechanisms, either of which may produce an increase in activity in a brain area: functional engagement in the task vs. release of suppression.

## Introduction

Research in cognitive neuroscience has implicated cortical regions near the right temporo-parietal junction (rTPJ) in a broad variety of tasks ranging from social interactions (Saxe and Powell, 2006) to attentional interactions with inanimate, visuo-spatial stimuli (Corbetta and Shulman, 2002; Corbetta et al., 2008). The central issue for this paper is how we may best account for observations of rTPJ involvement in attention and social processing.

### Anatomical and functional ambiguity at the rTPJ

The rTPJ does not have a distinct anatomical marker, but is considered to lie at the conjunction of the posterior superior temporal sulcus, the inferior parietal lobule and the lateral occipital cortex (Corbetta et al., 2008). This region of cortex has an unusually high degree of inter-individual variability in gross anatomical structure, as revealed both by careful anatomical observation (Ono et al., 1990) and quantified measures (Van Essen, 2005). Work on the cytoarchitecture of this region reveals substantial individual variation both in the size of functional regions and in the relationship between cytoarchitectonic borders and macroanatomical landmarks (Caspers et al., 2006). These factors make precise localization of functional regions near rTPJ identified using fMRI and PET challenging. A number of distinct anatomical labels have been used in the literature, including rTPJ, angular gyrus (AG), inferior parietal lobe, supramarginal gyrus (SMG), posterior temporal cortex and posterior superior temporal sulcus. These labels are not always used consistently; hence they cannot be relied upon to discriminate one functional region from another. Here we focus on a putative functional division between more posterior TPJ regions, including the AG, and more anterior TPJ regions, including the SMG.

### Attention and the rTPJ

The rTPJ is thought to play a role in reorienting attention to behaviorally salient stimuli. The exact requirements for a stimulus to be considered salient remain unclear (Frank and Sabatinelli, 2012), however, the area has been shown to respond to distractors that share features with the target stimulus (Indovina and Macaluso, 2007) or are spatially informative of a targets' location (Geng and Mangun, 2011). Regions near rTPJ show increased activity in response to breeches of expectation as well as identification of the target stimulus itself (Corbetta and Shulman, 2002). The most prominent theory integrating the rTPJs' function with other attentional processes suggests the area belongs to a right lateralized ventral attention network (VAN), composed of the TPJ, the middle and inferior frontal gyrus, frontal operculum, and anterior insula (Corbetta et al., 2008).

Current theory (Corbetta et al., 2002, 2008) suggests the VAN, specifically the rTPJ, plays the role of detecting unexpected but behaviorally relevant stimuli, and acts as a circuit breaker for the dorsal attention network (DAN). The DAN (Corbetta et al., 1998; Fox et al., 2005, 2006) is comprised of the intraparietal sulcus (IPS), superior parietal lobule, and the frontal eye fields (FEF) and is thought to be involved in top-down attentional processes. The DAN maintains visuo-spatial information with regards to the current task-defined goals, such as in response to a directional cue, while the VAN remains inhibited until a target or salient distractor is presented, at which point activity in the VAN interrupts the maintenance of attention in the DAN in order to reorient attention (Corbetta et al., 2002, 2008). Within the context of the VAN, the rTPJ has been most studied using variations on two tasks: oddball and Posner cue paradigms.

The standard oddball paradigm presents less frequent stimuli against a stream of frequent stimuli. The key feature is the novel/rare nature of the oddball targets compared to the typical or standard/frequent nature of the baseline stimulus. Visual stimuli are typically presented sequentially at a central fixation point (Bledowski et al., 2004) and in auditory tasks the stimuli are typically presented through headphones in both ears simultaneously (Stevens et al., 2005), although exceptions exist (Linden et al., 1999). As a result, the extent to which the task elicits spatial reorienting is often limited. In most instances participants are instructed to respond with a button press (Downar et al., 2001, 2002; Kiehl et al., 2005) or keep a mental count (Linden et al., 1999) of the number of target stimuli presented in the visual, auditory, and tactile sensory modalities (Linden, 2005).

The Posner cue-type experiment triggers the reorienting of attention in response to invalid cues. During the task the participant is presented with a central cue that more often than not predicts the location of a target stimulus. During invalid trials, the participant is cued to a different location than that of the target stimulus, necessitating a spatial reorienting of attention toward the target. The goal of the task is to detect the target stimulus and respond with a button press upon detection (Macaluso et al., 2002). The task has been studied in the visual (Corbetta et al., 2002) and auditory (Mayer et al., 2009) sensory modalities.

The oddball and Posner cue-type designs both involve the detection of unexpected (low frequency) task-relevant stimuli. Since this is a hypothesized function of the VAN, the co-localization of activations associated with both paradigms is consistent with theoretical accounts of the VAN. However, these tasks also differ in at least one important respect. Posner cue-type tasks require the reorienting of attention from one spatial location to another to respond to invalid trials. In contrast, oddball tasks don't require the participant to break their current focus of attention and make a spatial shift to a new location when a low frequency stimulus is presented.

### Social cognition and the rTPJ

The rTPJ has also been strongly implicated in social reasoning, specifically theory of mind (ToM) tasks. ToM refers to the ability to understand the intentions of a conspecific, i.e., to predict their actions through the attribution of beliefs and desires (Gallagher and Frith, 2003). ToM studies typically involve short stories followed by questions about the beliefs of one of the protagonists (Gallagher et al., 2000; Saxe and Powell, 2006) or the attribution of intentions to characters depicted in a comic strip (Vollm et al., 2006). The ToM condition is typically contrasted with stories describing human activity without the need for mental state attributions, such as outdated physical representations (Perner et al., 2006).

The rTPJ is part of a larger network of regions which is consistently activated by a variety of social cognition tasks which involve thinking about internal mental states, often referred to as the mentalizing network (Ochsner et al., 2004; Amodio and Frith, 2006; Saxe et al., 2006; Van Overwalle, 2009; Denny et al., 2012; Mars et al., 2012b; Schilbach et al., 2008, 2012). The regions which are most consistently associated with mentalizing are the rTPJ, the medial parietal/posterior cingulate cortex (MP/PCC) and the dorsal medial prefrontal cortex (dMPFC). There is evidence that the these medial mentalizing regions play a relatively general role in social cognition, including emotion processing and introspection (Schilbach et al., 2012), whereas the function of the rTPJ appears to be more specific to the attribution of beliefs and intentions to others (Saxe and Powell, 2006; Saxe et al., 2006).

### Relationship between attention and social cognition in the rTPJ

The current literature remains unsettled as to the extent the locus of activity at the rTPJ for mental state attribution coincides with the locus of activity at the rTPJ region involved in attentional processes. Mitchell (2007) found no topographical distinction between either process at the group or individual level of analysis. A meta-analysis published by Decety and Lamm (2007) found overlapping yet significantly different areas recruited for social and reorienting processes. Decety and Lamm's interpretation of these findings focuses on the overlap. This is curious, since meta-analytic investigations can statistically support the claim that two conditions have distinct spatial profiles, but cannot directly speak to the issue of whether two regions do or do not have functional overlap^1^. Nonetheless, these researchers explain these findings by noting there may be similarities between the process involved in reorienting spatial attention and reorienting to another person's point of view (Decety and Lamm, 2007; Mitchell, 2007; Corbetta et al., 2008). In contrast, Scholz et al. (2009) found evidence of distinct activation peaks associated with ToM and attention reorienting, using both group and individual level analyses^2^. These authors resist the view that attention reorienting and ToM tasks share a common neural or psychological mechanism.

An important finding from work in resting state functional connectivity (rs-fcMRI) is the observation of negative correlations between cortical networks. Fox et al. (2005) identify two anticorrelated networks: the default mode network (DMN) and the task positive network (TPN). The DMN includes a region near rTPJ, the AG. The TPN overlaps the DAN and a second network called the fronto-parietal control network (FPCN) (Vincent et al., 2008)^3^. The TPN also includes a region near the rTPJ, the SMG (Fox et al., 2005; Jack et al., 2012). Research on the relationship between social and non-social processes in the brain suggests these antagonistic networks support two distinct cognitive domains. The opposing domains hypothesis holds that the mutually inhibitory relationship between the DMN and TPN reflects a cognitive tension between social cognition (including mentalizing and introspection) and non-social cognitive processes (typically recruited by attention demanding non-social tasks) (Jack et al., 2012). These findings suggest not just that there are at least two distinct regions near rTPJ, but also that they are in tension with each other. This claim is supported not only by resting state functionally connectivity analysis, but also by the finding that the same regions are activated and suppressed (relative to a resting baseline) by different task conditions (Jack et al., 2012). The task-induced activation and deactivation of these regions is important to note, because this evidence cannot be explained away as a potential artifact of methods commonly used in functional connectivity analyses (Murphy et al., 2009). Critically, a broad range of evidence now supports the view that the maintenance of externally-oriented attention in non-social tasks suppresses activity in the DMN below resting levels (Raichle and Snyder, 2007). It follows from this that the breaking of attention may give rise to a relative increase in activity in regions associated with social cognition, even in the absence of any social processing demands and purely as a result of the termination of suppression—allowing activity to return to resting levels.

rs-fcMRI has also been used as a data-driven tool to identify the borders of distinct functional regions on the basis of changes in connectivity. Initial work on this application indicates considerable variability in the degree to which clear boundaries between regions can be defined (Cohen et al., 2008), however, some areas contain very clear boundaries between contiguous regions with highly disjoint patterns of functional connectivity. One such boundary occurs in the TPJ, between the AG and SMG, in the immediate vicinity of activation foci associated with ToM tasks and with the VAN. These findings support the existence of two distinct functional networks, including a more posterior region incorporating the AG and a more anterior region incorporating the SMG, which are contiguous at the TPJ (see Figure 3 in Cohen et al., 2008). The existence of more than one region in this area is also supported by work in a distinct modality, diffusion tensor imaging, which identifies distinct regions near the rTPJ using tractrography–based parcellation (Mars et al., 2012a).

### An alternative account

The opposing domains hypothesis holds that regions involved in non-social attentional processing and social cognition are not only distinct, but also tend to suppress each other. How might this theory account for observations of the rTPJ's involvement in both attention and social processing? We suggest extending the opposing domains hypothesis with an additional auxiliary hypothesis: the breaking of attentional set that occurs during reorienting of attention leads to an increase in activity in social regions as a result of the release of suppression associated with the maintenance of focused attention. If both the opposing domain hypothesis and this auxiliary hypothesis are correct, then several predictions follow: (1) There should be distinct loci of activation associated with processes which are clearly social in nature (e.g., ToM tasks) and processes which are clearly non-social (e.g., detection of a non-social target, as occurs in oddball tasks). (2) Invalid trials in Posner-cue type experiments should lead both to an increase in activity in social regions (associated with release of suppression during reorienting) and an increase in activity in non-social regions (associated with detection of a non-social target).

The opposing domains account suggests distinct rTPJ areas are involved in social and attentional processing. Why might researchers have struggled to clearly distinguish between these putatively distinct but adjacent areas? We suggest that the region's inconsistent structural organization and variations across experimental paradigms have resulted in the misattribution of contiguous regions' response profiles to a single region. The response profile of the rTPJ, in the context of the VAN, may be falsely informed by fMRI findings that fail to account for the strong negative correlation, observed both in resting connectivity and due to tasks, between separate areas at the rTPJ. BOLD changes associated with reorienting may reflect the sum of two independent effects which occur in contiguous regions effectively simultaneously (given the temporal resolution of fMRI). The first is activation above resting baseline of the SMG associated with the detection of a low-frequency task-relevant stimulus. The second is release of deactivation in the AG, possibly only a recovery to baseline levels, which may in some paradigms be followed by a rapid return to a suppressed state due to processes involved in target detection (SMG activation) and/or re-engagement of attention (DAN activation). Although these two putative effects would reflect very different cognitive mechanisms, they may nonetheless produce similar event-related responses in immediately contiguous regions.

If this account is correct, then the “circuit breaker” function which VAN theory attributes to the rTPJ may be best explained by the posterior TPJ's (including the AG) involvement in social cognition, a type of processing which is in competition with focused attention. Such an account would still suggest a possible “circuit breaker” role for the posterior TPJ, however, this role would likely be non-specific in nature, involving a tendency to suppress attentional processes in general rather than communicating specific information that might inform the re-orienting of attention. This account holds that the anterior TPJ (including the SMG), in contrast to the posterior TPJ (including the AG), is directly involved in attentional processes.

### Summary of key hypothesis

The key hypothesis we propose here, and marshal evidence to support, is as follows: Reorienting (unlike oddball) paradigms require the participant to break their attentional set i.e., on invalid trials the participant must release sustained focused attention from its cued location to complete the task. The maintenance of focused attention is (one of) the cognitive process that tends to suppress DMN regions (while activating attention regions). When focused attention is broken, this suppression is (usually only temporarily) lifted. This causes activity in the posterior TPJ (e.g., AG) to increase relative to its suppressed state, just as happens when a compressed spring is released.

While this hypothesis is novel and tentative in the context of attention reorienting tasks, there is prior evidence which broadly supports this “compressed spring” model of DMN network activity. There is clear evidence that DMN regions are more suppressed for higher effort non-social tasks, and that there is return to baseline when participants disengage, either because the task finishes or because of mind-wandering (McKiernan et al., 2003; Mason et al., 2007). In addition, there is evidence of a “rebound” effect, such that DMN activity is greater during resting periods the more it has been suppressed by a preceding working memory task (Pyka et al., 2009). We hypothesize that the sudden breaking, and subsequent refocusing, of attention that occurs in reorienting tasks produces a similar pattern, but on a shorter timescale. That is, reorienting produces a transient release of suppression whose BOLD time course looks similar to that of an above-baseline event related response.

While this hypothesis is tentative, it nonetheless raises questions about the view that the AG is involved in attentional reorienting in the manner envisaged by VAN theory. In addition to having implications for VAN theory, this idea has quite broad implications for the interpretation of neuroimaging findings. The usual inference that is made from the observation that an area increases in activity concomitant with a task event is that the area plays a direct functional role in the task-related cognitive processes that occur at that moment. This is the basic logic of cognitive subtraction (Price and Friston, 1997). However, this logic has already been implicitly acknowledged as incorrect for cases where an increase in activity can be more simply explained by a decrease in suppression (McKiernan et al., 2003; Mason et al., 2007). VAN theory focuses on a region which, similar to other DMN regions, is typically deactivated compared to rest during task performance (Shulman et al., 2007). VAN theory interprets activation of this region following the well-established and intuitive logic of cognitive subtraction. Our provocative suggestion is that this logic fails to apply. Specifically, we suggest that transient increases in activity near the AG have been incorrectly attributed to that region playing an active role in attention reorienting, when the observed effect is really due to the transient release of suppression of that region^4^.

### Experimental design

To test our alternative account of rTPJ involvement in attention and social cognition, we sought to localize and investigate the functional connectivity of regions associated with the detection of task-relevant infrequent stimuli, the attribution of intentions to agents, and the reorienting of attention. To do this, we use formal meta-analytic methods to distinguish the localization of activations associated with oddball, ToM and reorienting paradigms. Of particular significance is that, unlike a prior formal meta-analysis which investigated attention and social processes in rTPJ (Decety and Lamm, 2007), we distinguish oddball from reorienting tasks. We predict that oddball paradigms will preferentially recruit the anterior TPJ (e.g., SMG), ToM tasks will preferentially recruit the posterior TPJ (e.g., AG), and reorienting will tend to be localized between the AG and SMG. Next, we examine functional connectivity associated with these distinct foci. In accordance with the opposing domains hypothesis we predict very different cortical networks will be associated with ToM and oddball seeds. The reorienting seed is predicted to lie on the border between these networks, and hence correlations with this seed should reflect some combination of signals associated with the other two seeds.

## Materials and methods

### Literature search and coordinate selection

The research articles used as a source of foci for the meta-analyses were identified in two ways. First, we gathered papers referenced in Decety and Lamm's formal meta-analysis (2007), as well as Corbetta and Shulman's (2002), and Corbetta, Patel and Shulman (2008) reviews. Second, additional papers were identified by performing a search on Google Scholar using the terms “fmri” or “pet”; and “reorienting,” “posner,” “oddball,” “target detection,” or “ToM.”

Once a database of 50 potentially relevant papers was identified, each paper was categorized as containing either a ToM, attention reorienting, or target detection task. ToM tasks were defined as reasoning about beliefs, intentions, or thoughts. Foci of interest contrasted tasks requiring the attribution of mental states to matched tasks that did not require the participant to consider others' beliefs or intentions. Attention reorienting tasks were defined as redirecting attention toward a target stimulus after a breach of expectation. Foci of interest contrasted trials when participants had to redirect attention after being misinformed about the upcoming target stimulus' location to trials when participants were correctly informed. Target detection tasks were defined as the presentation of a distinct and infrequent stimulus during a stream of frequent stimuli. Foci of interest contrasted trials when participants encountered an oddball to non-oddball trials.

Rather than filtering out papers based on a reported coordinates' proximity to idealized rTPJ coordinates as in a prior metanalysis (Decety and Lamm, 2007), foci tables containing analyses that reflected a given task definition were all included in the meta-analyses. All of the foci from an analysis were extracted from a paper and reported in stereotactic coordinates (*x, y, z*). If the coordinates were reported in the Montreal Neurological Institute space, they were converted to the Talairach and Tournoux (TAL) space using the Brett transformation (Brett, 1999).

### Meta-analyses

Separate meta-analyses were performed to localize activation for each task using activation likelihood estimation (Eickhoff et al., 2009), with a full-width-at-half-maximum (FWHM) of 10 mm, *p*-value threshold of *p* < 0.004, and a false discovery rate (FDR) threshold of *q* = 0.05. In addition, differences in activation between the three tasks were computed using difference maps (Laird et al., 2005), using 5000 permutations. The thresholded ALE maps from both analyses were visualized on a fiducial representation of a standardized brain atlas (PALS-B12 human atlas) using Caret version 5.612.

### Resting state functional connectivity analyses

For each task, the results of the meta-analyses were visualized in Caret and the centres of activation near the rTPJ were identified and used as seeds for three separate resting state functional connectivity analyses. Table 1 lists the coordinates used as seeds for the analyses. Resting state data was retrieved from the public database NITRC on February 15, 2010. Two data sets were used: Beijing_Zang (Zang, Y. F.; *n* = 198 [76M/122F]; ages: 18–26; *TR* = 2; no. of slices = 33; no. of timepoints = 225) and Cambridge_Buckner (Buckner, R. L.; *n* = 198 [75M/123F]; ages: 18–30; *TR* = 34; no. of slices = 47; no. of timepoints = 119). The total combined number of subjects was 396 (245 female), aged 18–30 (mean age 21.1). The data was aligned to 711–2B atlas space. All methods were identical to those reported by Fox et al. (2005, 2006, 2009; Jack et al., 2012) and similarly employed a global gray matter regressor, except that statistical contrasts used a random effects method (Jack et al., 2012), and the resulting statistical images were whole brain corrected for multiple comparisons (*z* > 3, *n* = 17). Contrasts either used one fisher-*z* transformed correlation image per subject entered into a single sample *t*-test, or two such images corresponding to the two seeds entered into a paired *t*-test.

**Table 1:** **Connectivity analysis coordinates**.

	***x***	***y***	***z***
Reorienting	54	−47	21
Target detection	55	−37	18
ToM	50	−55	23

Coordinates used as seeds for each task in the resting state connectivity analyses.

## Results

### Meta-analyses

The studies used in the primary meta-analyses are listed in Tables 2–4. In total, the reorienting category contained 14 papers (139 foci), 12 papers (199 foci) were in the oddball category, and 12 papers (104 foci) were in the ToM category.

**Table 2:** **Target detection meta-analysis studies**.

**Authors**	**Analysis**
Bledowski et al., 2004	Regions activated during target condition vs. baseline
Braver et al., 2001	Regions showing consistent response to low-frequency events in conjunction analyses
Downar et al., 2001	Relevant stimulus changes minus irrelevant stimulus changes
Downar et al., 2002	Greater response to novel than familiar stimuli across all sensory modalities
Fichtenholtz et al., 2004	Attentional targets (shape oddballs and emotional pictures)
Kiehl et al., 2005	Detection of target stimuli minus standard stimuli
Kiehl et al., 2001	Target stimuli minus non-target baseline condition
Liebenthal et al., 2003	Peaks of BOLD activation correlated with the magnitude of the ERP negativity during the MMN range
Linden et al., 1999	Response to targets vs. response to non-tragets
Melcher and Gruber, 2006	Color-oddballs vs. oddball control
Stevens et al., 2005	Right hemisphere minus left hemisphere; oddball detection
Watkins et al., 2006	Singleton trials compared with no singleton trials

**Table 3:** **Reorienting meta-analysis studies**.

**Authors**	**Contrast**
Arrington et al., 2000	Invalid minus valid
^*^Astafiev et al., 2006	Peak TPJ activation in Validity × Time
Corbetta et al., 2002	Invalid minus valid
^*^Giessing et al., 2006	Validity main effect
^*^Giessing et al., 2004	Event and block-related validity effects
Indovina and Macaluso, 2007	Invalid minus valid
Kincade, 2005	Endogenous condition validity by time
^*^Konrad et al., 2005	Invalid minus valid (adults only)
Lepsien and Pollmann, 2002	Validity effects within SOA of 100 ms
Macaluso et al., 2002	Invalid minus valid
Mattler et al., 2006	Invalid minus valid
Mayer et al., 2009	Invalid > valid (100 ms SOA)
Mayer et al., 2006	Invalid minus valid
Mayer et al., 2007	Invalid > valid (100 ms SOA)
Mitchell, 2007	Invalid minus valid
Natale et al., 2009	Invalid minus valid endogenous cues
Thiel et al., 2004	Invalid minus valid trials
Vossel et al., 2006	Reorienting in the 90% validity condition

* Denotes additional papers included in the secondary meta-analysis.

**Table 4:** **Theory of mind meta-analysis studies**.

**Authors**	**Contrast**
^*^Aichorn et al., 2009	False belief > photo (question)
^*^Abraham et al., 2010	Belief-questions > control-questions and desire-questions > control-questions
^*^Bahnemann et al., 2010	ToM judgments minus appearance judgments
^*^Bruneau et al., 2012	ToM localizer
^*^Dohnel et al., 2012	Sally Anne task (true and false ToM minus reality)
Fletcher et al., 1995	ToM stories vs. Physical stories
Gallagher et al., 2000	ToM vs. non-ToM stories
Gobbini et al., 2007	False belief stories vs. physical belief stories
^*^Hartwright et al., 2012	False belief minus false photograph
Hynes et al., 2005	Cognitive PT minus Control
^*^Jenkins and Mitchell, 2010	Mentalizing scenarios > non-social scenarios
^*^Kobayashi et al., 2008	ToM > physical (both japanese and english language groups)
^*^Kobayashi et al., 2006	ToM compared with non-ToM-conjunction among language groups
^*^van der Meer et al., 2011	ToM high inhibition minus fixation
Mitchell, 2007	Tom minus attention cueing task
Perner et al., 2006	False belief vignettes minus photo vignettes
^*^Rabin et al., 2009	ToM photo minus autobiographical memory photo
Ruby and Decety, 2003	3rd person minus 1st person
^*^Samson et al., 2008	ToM cartoons minus non-ToM cartoons
Saxe and Kanwisher, 2003	ToM inference minus mechanical inference
Saxe and Powell, 2006	False belief minus false photograph
Saxe et al., 2006	ToM reference experiment
^*^Veroude et al., 2012	Others vs. self (females only)
Vollm et al., 2006	ToM minus physical causality one character
^*^Wolf et al., 2010	Social minus physical inference (multiple choice and silent)
Young et al., 2007	Belief minus photo
^*^Young et al., 2010	Mental > physical sentences
^*^Zaitchik et al., 2010	Belief sentences > control sentences

* Denotes additional papers included in the secondary meta-analysis.

**Figure 1:**
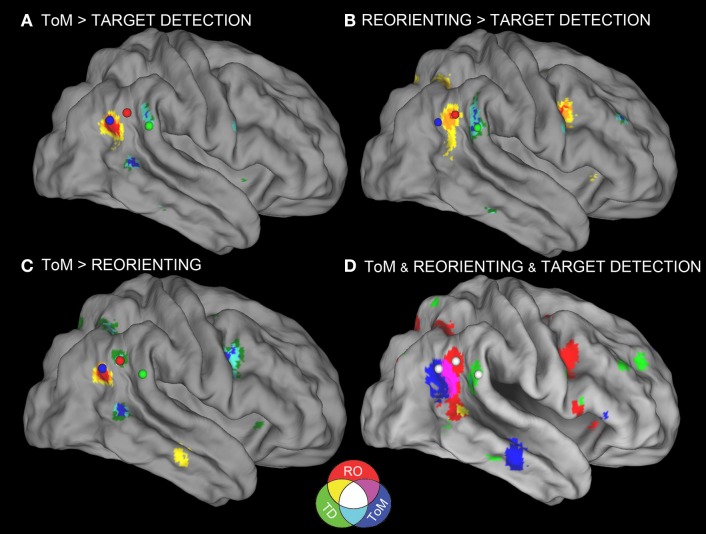
**Meta-analyses results with connectivity seeds**. Results from the difference maps comparing **(A)** ToM and target detection, **(B)** reorienting and target detection, **(C)** ToM and reorienting tasks. All three tasks show regions near the rTPJ that survived the pairwise difference maps. **(D)** Results from the individual meta-analyses. Each panel shows the peaks of activation clusters near rTPJ in the analysis shown in Figure 2. ToM (50, −55, 23), reorienting (54, −47, 21), and target detection (55, −37, 18). Note: color key applies to activations in **(D)** and foci colors in **(A–C)**, activation in **(A–C)** are colored based on *T*-statistics. This figure reflects the secondary extended meta-analysis (see results).

In response to a reviewer concern that the meta-analysis accurately represented each category, a secondary, *post hoc* meta-analysis was conducted including foci from an additional four reorienting and 16 ToM papers. A total of 18 reorienting papers (169 foci) and 28 ToM papers (239 foci) were used in the secondary analysis. Papers used in the secondary meta-analysis are listed and indicated in Tables 2–4. Figure 1 shows the results from this secondary extended meta-analysis instead of the primary analysis. The results were highly consistent, such that the seed regions originally identified by identifying peak significance did not need to be altered (Figure 1). The principle difference between the two meta-analyses was that the secondary analysis produced more extended areas of significance in the expanded categories.

Figure 1D displays the results of the three single-condition analyses. Each of the three conditions shows areas of activation unique to each task (see figure description for peaks of activation; Table 5 for whole-brain peaks of activation). The ToM and reorienting region-of-interest (ROI) near the rTPJ show some overlap (purple area), with the ToM ROI extending more posterior at the AG and the reorienting ROI extending more anterior. While the peak of the reorienting ROI lay dorsal to the ToM ROI, the reorienting ROI extended in a dorsal-ventral direction such that it clearly separated a posterior TPJ region (including the AG) from an anterior TPJ region (including the SMG). Note the clearly distinct peak activation region at the rTPJ for the target detection ROI, located more anterior at the SMG compared to both the ToM and reorienting ROIs. Figures 1A–C displays the results of the difference maps. All three comparisons resulted in distinct areas of peak activation for each task near the rTPJ, conforming to the same spatial distribution suggested by the initial meta-analyses. The peaks of activation clusters for each difference map from the primary analysis are listed in Table 6.

**Table 5:** **Meta-analyses results**.

**Category**	**Area**	**Ceneter (TAL)**
Target detection	L medial frontal gyrus	(0.21, 6.66, 44.4)
	R superior temoral gyrus	(55.24, −37.47, 17.68)
	L transverse temporal gyrus	(−53.09, −24.14, 12.42)
	L postcentral gyrus	(−34.26, −40.5, 58.21)
	R thalamus	(7.46, −15.03, 7.84)
	L postcentral gyrus	(−37.76, −24.58, 55.43)
	R middle temporal gyrus	(52.69, −25.11, −11.65)
	L cerebellum	(−25.54, −59.95, −30.56)
	R inferior frontal gyrus	(48.98, 6.48, 21.1)
	L inferior parietal lobule	(−57.01, −38.69, 25.89)
	R precentral gyrus	(41.87, 9.58, 6.36)
	R cerebellum	(17.26, −49.15, −27.23)
	R superior frontal gyrus	(20.04, 45.89, 30.96)
	L thalamus	(−11.39, −19.29, 6.59)
	R middle temporal gyrus	(54.91, −53.38, 1.45)
	L superior frontal gyrus	(−36.53, 36.63, 27.94)
	L superior temporal gyrus	(−46.3, 10.73, −6.03)
	L superior temporal gyrus	(−53.82, −6.52, −4.32)
	L middle temporal gyrus	(−58.22, −56.83, 3.1)
Reorienting	R supramarginal gyrus	(54, −47.27, 20.51)
	L precentral gyrus	(−43.51, 3.52, 30.65)
	R inferior frontal gyrus	(41.01, 9.3, 31.32)
	L superior frontal gyrus	(−0.54, 9.68, 53.26)
	R premotor cortex 6	(28.84, −2.38, 55.04)
	R precuneus	(11.66, −65.88, 44.92)
	L inferior parietal lobule	(−36.35, −45.52, 41.09)
	R inferior parietal lobule	38.11, −45.99, 45.29
	L middle frontal gyrus	(−29.54, −5.41, 53.56)
	L precuneus	(−11.62, −66.87, 47.38)
	R cerebellum	(17.41, −57.23, −33.62)
	R superior temporal gyrus	(41.08, −45.25, 18.5)
	L cerebellum	(−9, −38.61, −41.39)
	L superior temporal gyrus	(−56.98, −45, 12.64)
	R inferior frontal gyrus	(48.39, 13.58, 9.13)
	R superior occipital gyrus	(34.04, −78.14, 30.68)
	R insula	(32.9, 22.88, −0.07)
	R precuneus	(31.32, −66.21, 32.08)
	L precuneus	(−6.87, −72.25, 34.58)
Theory of mind	L superior temporal gyrus	(−49.02, −58.44, 22.05)
	R superior temporal gyrus	(50.18, −54.58, 22.51)
	L cingulate gyrus	(−1.26, −54.89, 26.65)
	L medial frontal gyrus	(−3.12, 51.22, 13.82)
	R medial frontal gyrus	(2.91, 51.58, 33.85)
	R middle temporal gyrus	(58.64, −16.97, −13.44)
	L middle temporal gyrus	(−56.17, −25.21, −8.62)
	R superior frontal gyrus	(8.64, 19.56, 55.45)
	L inferior temporal gyrus	(−49.79, −4.8, −28.86)
	L superior frontal gyrus	(−17.47, 46.57, 37.76)
	R putamen	(24.84, 3.96, −8.05)
	L parahippocampal gyrus	(−24.58, −2.4, −16.89)

Coordinates of clusters produced by the primary meta-analyses. Anatomical labels produced by GingerALE.

**Table 6:** **Difference maps results**.

**Contrast**	**Ceneter (TAL)**	**Category**	**Subjects represented (Category)**	**Authors**	**Sensory modality**	**rTPJ mentioned**
REATTN-ODATTN	(55.02, −31.98, 23.81)	ODATTN	42%	Linden et al., 1999	auditory/vision	20%
				Downar et al., 2002	vision/auditory/tactile	
				Kiehl et al., 2005	auditory	
				Liebenthal et al., 2003	auditory	
REATTN-ODATTN	(53.3, −47.36, 28.86)	REATTN	21%	Mitchell, 2007	vision	100%
				Vossel et al., 2006	vision	
TOM-ODATTN	(55.63, −37.65, 18.44)	ODATTN	54%	Bledowski et al., 2004	vision	33%
				Kiehl et al., 2001	auditory	
				Linden et al., 1999	auditory/vision	
				Downar et al., 2002	auditory/vision	
				Downar et al., 2001, 2002	vision/auditory	
				Kiehl et al., 2005	auditory	
				Liebenthal et al., 2003	auditory	
TOM-ODATTN	(49.61, −54.86, 22.74)	TOM	85%	Saxe et al., 2006	vision	89%
				Mitchell, 2007	vision	
				Young et al., 2007	vision	
				Saxe and Powell, 2006	vision	
				Fletcher et al., 1995	vision	
				Hynes et al., 2005	vision	
				Perner et al., 2006	vision	
				Saxe and Kanwisher, 2003	vision	
TOM-REATTN	(60.48, −36.52, 19.64)	TOM	70%	Mitchell, 2007	vision	75%
				Young et al., 2007	vision	
				Fletcher et al., 1995	vision	
				Hynes et al., 2005	vision	
				Perner et al., 2006	vision	
				Saxe and Kanwisher, 2003	vision	
TOM-REATTN	(60.48, −36.52, 19.64)	REATTN	61%	Mitchell, 2007	vision	88%
				Macaluso et al., 2002	vision/tactile	
				Vossel et al., 2006	vision	
				Mayer et al., 2006	auditory	
				Corbetta et al., 2002	vision	
				Mayer et al., 2009	auditory	
				Mattler et al., 2006	auditory/vision	
				Natale et al., 2009	vision	

Results from the difference maps from the primary meta-analysis. Centres of activation as reported by GingerALE for each contrast listed with papers containing foci that fell within the areas of activation. Note that a foci does not have to lie within a cluster to significantly contribute to the cluster. "Subjects represented' is the percent of subjects from the papers within the significant cluster over the total amount of subject in the given task category. “rTPJ mentioned” is the percent of papers specifically implicating the rTPJ within the significant clusters. REATTN, reorienting; ODATTN, target detection; TOM, theory of mind.

These findings support our hypotheses that the detection of infrequent behaviorally-relevant stimuli is associated with peak activation in the anterior TPJ (SMG) that attributing intentions to others is associated with a distinct locus of peak activation in the posterior TPJ (AG), and that tasks involving spatial reorienting demonstrate peak activation at points intermediate between these areas.

### Resting state functional connectivity analyses

**Figure 2:**
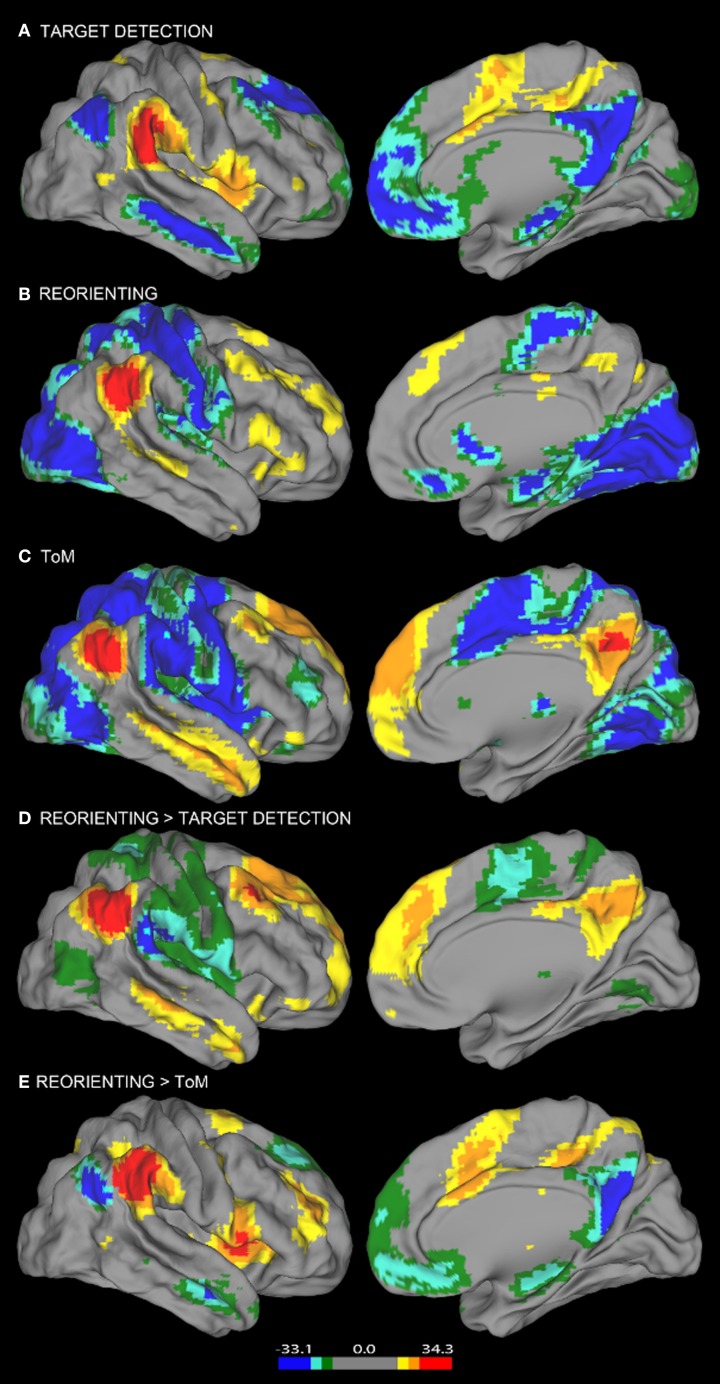
**Resting state connectivity results**. Results from the resting state connectivity analyses for each seed showing distinct patterns of connectivity for the **(A)** target detection, **(B)** reorienting, and **(C)** ToM seeds. The target detection seed shows a positive relationship with the TPN and a negative relationship with areas of the DMN. The ToM seed shows the opposite pattern, a positive relationship with the DMN, and a negative relationship with TPN areas. Results from the resting state connectivity contrasts showing the comparison of **(D)** reorienting and target detection connectivity and **(E)** reorienting and ToM connectivity. The contrast shown in **(D)** yields a pattern of connectivity highly similar to the ToM seed connectivity **(C)**, while the contrast shown in **(E)** yields a pattern highly similar to the target detection seed connectivity **(A)**. Left hemisphere connectivity patterns were very similar to right hemisphere connectivity patterns.

Figures 2A–C displays the results of the resting state connectivity analyses.

**Figure 3:**
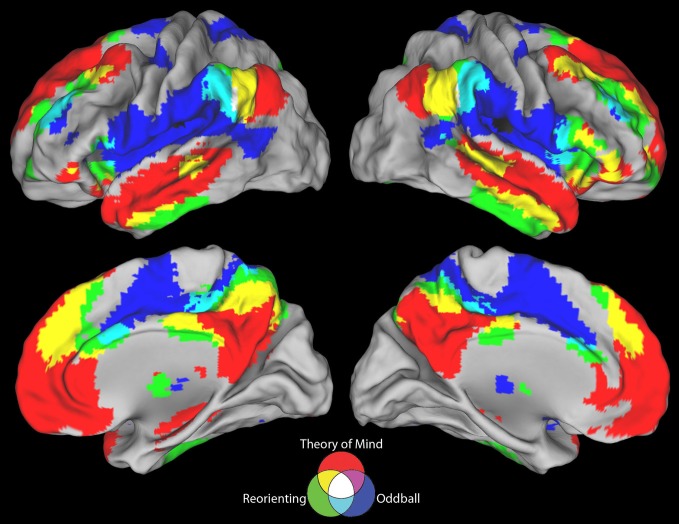
**Positive connectivity results for all three seeds**. The ToM and target detection seeds demonstrate a complete lack of overlap between their positive resting state correlation patterns (purple areas). All three seeds show minimal overlap in positive connectivity (white areas).

**Figure 4:**
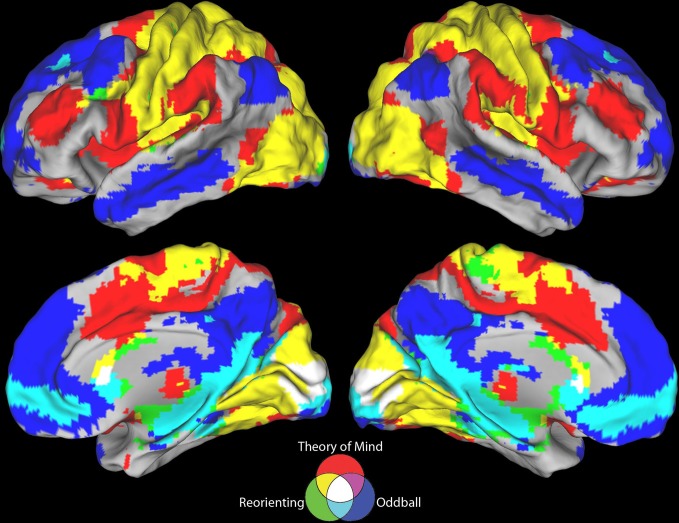
**Negative connectivity results for all three seeds**. The ToM and target detection seeds demonstrate a complete lack of overlap between their negative resting state correlation patterns (purple areas). All three seeds show minimal overlap in negative connectivity (white areas).

Consistent with our view that regions supporting ToM (e.g., AG) and regions supporting target detection (e.g., SMG) have distinct functional roles, the ToM and target detection ROIs show very different patterns of resting connectivity. There was a complete absence of overlap in either their positive or negative connectivity patterns (a direct comparison is illustrated in Figures 3, 4). Consistent with our claim that the ToM region is part of the DMN the ToM seed shows positive connectivity with the DMN, particularly MP/PCC and dMPFC regions associated with mentalizing. In addition, consistent with our claim that the ToM region has a reciprocal inhibitory relationship with the DAN, regions anti-correlated with the ToM seed show an excellent correspondence with the DAN as identified in prior publications (Fox et al., 2005, 2006).

The target detection seed demonstrates a positive relationship with the anterior insula, supplementary motor area, and anterior cingulate cortex; regions involved in saliency detection, effort, and task difficulty typically recruited during oddball tasks (Linden et al., 1999). Consistent with our claim that regions supporting target detection have a reciprocal inhibitory relationship with the DMN, regions anti-correlated with the target detection seed show an excellent correspondence with the DMN as identified in prior publications (Fox et al., 2005), including rTPJ, MP/PCC, and dMPFC regions specifically associated with mentalizing (Van Overwalle, 2009; Denny et al., 2012).

Similar to findings reported in Fox et al. (2006), our reorienting seed identified positively correlated regions in medial frontal gyrus, inferior frontal gyrus, a region in medial prefrontal cortex posterior to the dMPFC region previously mentioned, and anterior insula. Hence our positive connectivity pattern was broadly equivalent, however, the positive correlations we observed appeared relatively weaker, and we identified anti-correlations with DAN regions which were not observed by Fox et al. (2006).

Visual inspection of Figure 2B indicates that the reorienting seed demonstrates substantial overlap between both the positive and negative resting state correlation patterns of the ToM seed (see Figures 3, 4, yellow areas) and target detection seed (see Figures 3, 4, light blue areas). To further examine the hypothesis that the reorienting seed involves the combination of signals associated with the other seeds, we examined differences in connectivity between the reorienting seed and the two other seeds. If the reorienting seed corresponds to a region with a distinct functional connectivity pattern, then distinct regions should be observed which cannot be accounted for by the connectivity of the other seeds. However, this was not what we observed. Examining differences between the reorienting and target detection seeds (Figure 2D), we found a pattern very similar to that observed for the ToM seed (Figure 2C). In particular, no areas of positive connectivity were identified which could not be accounted for by hypothesizing that the reorienting seed involves the combination of signals from the ToM and target detection seeds. Examining differences between the reorienting and the ToM seeds (Figure 2E), we found a pattern very similar to that observed for the target detection seed (Figure 2A). There were two areas of positive connectivity which appeared greater than for the target detection seed, in anterior middle frontal gyrus, and inferior frontal/insula. However, these apparent positives could be accounted for by anti-correlations with the ToM seed. No areas of positive connectivity were identified which could not be accounted for by hypothesizing that the reorienting seed involves the combination of signals from the ToM and target detection seeds.

## Discussion

Our goal in this paper is to articulate an alternative account of the involvement of regions near the rTPJ in attention and social processing, and provide evidence which is more consistent with our account than with extant theory concerning the VAN.

### Challenges to VAN theory

Our findings are consistent with other findings which suggest there are at least two functionally distinct regions near rTPJ (Caspers et al., 2006; Cohen et al., 2008; Scholz et al., 2009; Mars et al., 2012a), and that these regions are part of two distinct networks which can be differentiated using rs-fcMRI (Fox et al., 2005; Cohen et al., 2008; Mars et al., 2012a) and by virtue of their differential engagement in attention demanding social and non-social tasks (Fox et al., 2005; Jack et al., 2012). We add to these prior observations by demonstrating that these distinct networks at the rTPJ correspond to distinct loci for target detection and ToM, using formal meta-analysis. These findings present three challenges to current theory concerning the VAN (Corbetta and Shulman, 2002; Corbetta et al., 2008).

First, contra Corbetta and Shulman (2002), our findings indicate that target detection has a distinct locus from reorienting. Current theory holds that oddball and reorienting paradigms both activate the VAN because both involve the detection of behaviorally relevant unexpected stimuli. However, we suggest this account oversimplifies reorienting of attention by equating it to a purely confirmatory process (i.e., target detection). A target is undoubtedly detected during invalid trials, but in addition, the preceding attentional set is broken and the locus of attention changed to the unexpected location. The existence of this additional process in the Posner cue-type design is supported by highly consistent findings of longer response times for invalid compared to valid trials (Corbetta et al., 2002; Hopfinger and Ries, 2005; Mayer et al., 2009). In contrast, there is no need to break attentional set in oddball paradigms. In accordance with our distinction between the two types of task, the meta-analysis identified two separate areas at the rTPJ for reorienting and target detection.

Second, contra Corbetta et al. (2008), our findings indicate that ToM paradigms recruit a neighboring but significantly distinct locus from reorienting and target detection. Our account can explain the seemingly contradictory findings of prior studies which have directly compared ToM and reorienting tasks. Importantly, both prior studies included analyses of individual participants, overcoming the problem of inter-individual differences at the rTPJ. Mitchell (2007) found no topographical distinction between either process, whereas Scholz et al. (2009) found evidence of distinct activation peaks associated with ToM and attention reorienting. These differences between the studies may be accounted for by differences in the methods of analysis, or by scanner resolution differences, as Scholz et al. suggest. Alternatively they may be due to differences in the designs of the reorienting paradigms, which are likely to have altered the relative balance of contributions made by the AG and SMG networks to the reorienting event-related signal^5^. In fact, even using high resolution imaging with regions defined in individual participants, Scholz et al. (2009) report modulation of the ToM area associated with reorienting and modulation of the reorienting area associated with ToM. This finding is difficult to account for on Scholz et al's own model, which holds the regions play wholly functionally distinct roles in reorienting and ToM. However, it is consistent with our view that ToM and target detection are functionally connected by virtue of a mutually inhibitory relationship (Jack et al., 2012). A meta-analysis published by Decety and Lamm (2007) also found a significant difference in peak activation location associated with social and attentional processes. Our results are consistent with theirs. However, they did not distinguish reorienting from target detection foci.

Third, contra Fox et al. (2006), our findings suggest that rs-fcMRI derivations of the VAN using a reorienting seed may result from the confounding of distinct signals. To allow a meaningful comparison, we used identical rs-fcMRI methods to the prior report (Fox et al., 2006). The only differences are that: our reorienting seed is based on a larger sample of reorienting foci which we analyzed using formal meta-analysis methods, our functional connectivity findings are derived from a considerably larger sample, we used random rather than fixed effects analysis methods, and we added the use of paired *t*-tests for the purposes of comparing connectivity associated with different seeds.

The contrast between the reorienting and target detection connectivity produced a correlation pattern almost identical to that of the ToM seed, whereas the contrast between the reorienting and ToM connectivity produces a correlation pattern almost identical to that of the target detection seed. The logic of our analysis is straightforward. If the reorienting seed corresponds to a distinct functional network, then the paired *t*-tests should have revealed evidence of connectivity to regions which could not be accounted for by correlations with the ToM and target detection seeds. We do not deny the possibility that there is a distinct functional network interposed between the AG and SMG, as suggested by some recent reports (e.g., Yeo et al., 2011). However, we do not believe that the methods used in these reports are able to clearly distinguish between correlations which arise due to the summing of signals from contiguous regions and correlations which genuinely reflect the existence of a distinct network. Further, we note very low confidence estimates for networks in this region (see Figures 8, 10 in Yeo et al., 2011). Since it is more parsimonious to assume two networks are present in this region, as opposed to three (Figure 7 in Yeo et al., 2011) or six (Figure 9 in Yeo et al., 2011), we suggest this should be the null hypothesis pending the development of independently validated methods that can unequivocally distinguish between these possibilities.

### Circuit breaking

VAN theory and our account are both consistent with a circuit breaking role for rTPJ regions which are suppressed during visual search. However, our account suggests a different type of circuit breaking. VAN theory holds that suppressed regions are involved in the filtering of unexpected stimuli and, when a task relevant unexpected stimulus is detected, send information about that stimulus to the DAN to guide the reorienting of attention (Shulman et al., 2007; Corbetta et al., 2008). Our account sees filtering and sending information about salient stimuli as potential functions of the anterior TPJ (e.g., SMG). The posterior TPJ (e.g., AG) is the primary locus of suppression, and is dedicated to tracking the intentions of perceived agents. Nonetheless, since the AG is in tension with the DAN, our account is consistent with its playing a more general circuit breaking role.

One possibility is that transient activation of the AG sends a non-specific reset signal to the DAN, akin to adding noise to a dynamic system so that it can settle into a new global minimum. However, we note that theoretical explanations proposing the role of the rTPJ as a circuit-breaker (Corbetta et al., 2008) lack confirmation of the area's purported beneficial role in resetting top-down influences from the DAN. The existing evidence shows increases in activity at rTPJ to be detrimental to target detection (Shulman et al., 2007), and a negative relationship between behavioral performance and a measure of the VAN's causal influence on the DAN (Wen et al., 2012). Research on the time course of the rTPJ and DAN, while not conclusive, suggests the rTPJ's activity follows transient activity in the DAN (DiQuattro and Geng, 2011); results contrary to the circuit-breaker hypothesis of rTPJ function. Instead, the anterior TPJ (SMG) may be involved in updating attentional sets by working in concert with the IFG, which in turn modulates activity in the DAN (Sridharan et al., 2007; DiQuattro and Geng, 2011; Vossel et al., 2012; Weissman and Prado, 2012). Hence, we remain neutral concerning the potential circuit breaking role of the posterior TPJ (e.g., AG), awaiting evidence which more clearly distinguishes the roles of these regions. An alternative to the circuit breaker hypothesis, which is equally consistent with our account, is that disruption of a suppressive signal that originates either in the DAN or a third region such as the IFG causes the posterior TPJ (e.g., AG) to be temporarily released.

Published maps of the VAN obtained using rs-fcMRI are variable. There are notable discrepancies between two papers with overlapping authors (Fox et al., 2006; Mantini et al., 2009), most notably with regard to whether or not anti-correlations are seen with the DMN, but also to regions of positive connectivity. One of the VAN maps coheres well with our SMG target detection map (Mantini et al., 2009), the other is more similar to our reorienting seed map (Fox et al., 2006). Our account can readily explain such discrepancies, which may result from small variations in the location of the seed near the border between discrete functional networks. However, another possible explanation is the presence of a third, more dorsal region at the rTPJ, in-between the AG and SMG. Recent work has emphasized the role of additional networks other than the VAN and DAN in attention (Petersen and Posner, 2012). One such network, the frontoparietal control network (FPCN), is involved in moment-to-moment aspects of executive control, often associated with cue-onset activity within trials, and includes an area more dorsal than the rTPJ node of the VAN. However, the extent to which this region is distinct from DAN (Dosenbach et al., 2008) and VAN (Dosenbach et al., 2006) areas near the rTPJ remains unclear. Outside of standard attentional control tasks, the FPCN is also hypothesized to support executive control in tasks that specifically recruit the DMN (Spreng et al., 2010). Spreng et al. (2012) argues that the network supports goal-directed cognition, whether it be social or visuo-spatial in nature, pointing to the mediatory connectivity profiles between the FPCN and DAN, as well as the FPCN and DMN, as evidence.

The overlap between our reorienting connectivity areas and the FPCN is unclear, nonetheless, our connectivity contrasts are potentially congruent with such an account. The FPCN's high degree of interconnectivity with both the TPN and DMN may be reflected in our finding that separately subtracting reorienting connectivity from AG and SMG connectivity leaves no regions left over that could not be explained by correlations with the AG and SMG seeds.

In summary, the number of attention networks has increased and evolved into a more complex account than simply the DAN and VAN (Corbetta and Shulman, 2002). Such a view is consistent with our account that reorienting is a complex process, however, our explanation does not require the addition of a network to explain reorienting-related activity at the rTPJ. If reorienting does rely on a third attentional network including a more dorsal rTPJ region, then our challenge to VAN theory would be restricted to the identification of a distinct region at the rTPJ involved in attention but dissociable from target detection (Corbetta and Shulman, 2011).

### Empirical limitations

We acknowledge limitations to our empirical findings. First, our meta-analytic findings rely on the anatomical alignment of studies conducted using different scanners whose images have been co-registered to different atlases. Given that our sample was of a reasonable size, these differences should have led an increase in randomly distributed noise and thus greater difficulty resolving distinct localizations. Nonetheless, the possibility of systematic error remains. Second, we have postulated that two factors contribute to reorienting responses. However, we have not directly manipulated these factors in order to establish this claim. Ideally, future work will employ high resolution imaging and paradigms that parametrically modulate these factors in order to distinguish their effects on different cortical areas. Third, we acknowledge that careful anatomical work suggests a number of distinct functional regions near rTPJ (Caspers et al., 2006) and that our group-based methods may have failed to capture important aspects of this fine grained structure. Although our work is at a similar anatomical resolution to work that has guided VAN theory, we acknowledge that higher resolution work on individual subjects may confirm the existence of a region specific to reorienting between the AG and SMG. Hence, our account of rTPJ involvement in reorienting in terms of the combination of signals from contiguous regions associated with two wide-scale functional networks may turn out to be wrong. In that case, our challenge to VAN theory would be restricted to noting the need to differentiate between regions involved in reorienting, target detection (Corbetta and Shulman, 2002) and ToM (Corbetta et al., 2008).

### Novel methodological claims

Our theoretical account of reorienting relies on two relatively novel claims. The first is that event-related BOLD effects with positive going waveforms can be attributed to the transient disengagement of suppression in a paradigm. The second is that positive connectivity maps derived from standard rs-fcMRI methods may, in some cases, fail to identify coherent functional networks. We acknowledge that further work is wanted to establish these claims. At the same time, we point to considerations which support the plausibility of these claims.

First, there is now a substantial body of work which establishes that activity levels of the default network can, in some cases, be best accounted for by the suppressive effect of task demands which are positively associated with functions instantiated in entirely distinct cortical networks (McKiernan et al., 2003; Mason et al., 2007; Buckner et al., 2008; Andrews-Hanna, 2011). If this view is accepted, it represents a relatively minor step to presume that the transient event-related release of these suppressive effects could give rise to a positive going BOLD waveform.

Second, we note that the methods of rs-fcMRI are relatively novel, and to date have only been partially validated. It has already been shown, both mathematically and in practice that they can produce artifactual results, particularly in relation to negative correlation maps (Murphy et al., 2009)^6^. Although we don't know of validated examples of spurious positive correlations, they are no less mathematically plausible. The unusually high degree of inter-subject variability in anatomy and functional organization at the TPJ (Van Essen, 2005; Caspers et al., 2006) further increases the potential for signals from neighboring but functionally distinct areas to be confounded when deriving rs-fcMRI maps of this area.

### Implications for theory

A natural assumption which has guided some prior accounts has been the view that attentional reorienting is an evolutionarily basic process which has been coopted to play a role in social cognition (Decety and Lamm, 2007; Corbetta et al., 2008). However, it is important to remember that the parsing of the cognitive operations involved in tasks is a complex and partially speculative process. Reorienting may not be a basic cognitive process, but may instead be a complex process which involves contributions from different regions with computationally distinct roles. Recent accounts of the evolution of the human cortex suggest that social processing demands have played an important role in the massive evolutionary expansion of cortex, which is evident from comparisons between humans and our nearest evolutionary neighbors. Our view is guided by this work, and suggests that some observations which propose a putative role for the rTPJ in attention may be best explained by an alternative hypothesis. Namely, the view that social processing is accomplished by basic cognitive processes which evolved specifically for that purpose, which are not only distinct from but also in tension with basic attentional processes.

While a synthesis of the attention literature lies beyond the scope of this paper, we suggest that some current ambiguities may be resolved by distinguishing between the functions of the anterior TPJ (e.g., SMG) and the posterior TPJ (e.g., AG). For example, a recent review on neglect proposes that the attentional deficits are a result of damage to VAN regions, disrupting communication between the left and right DANs (Corbetta and Shulman, 2011), however, the authors admit the neural mechanisms explaining interactions between the VAN and DAN are poorly understood. Research has demonstrated deficits in sustained attention in patients with posterior parietal cortex lesions (Malhotra et al., 2009) and target detection from TMS over the AG, not the SMG (Chambers et al., 2004). The AG region of the DMN has demonstrated abnormal functioning in patients with a variety of neurological disorders (Zhou et al., 2007; Broyd et al., 2009) as well as traumatic brain injuries (Bonnelle et al., 2011) characterized by low sustained attention. In light of our results, we suggest that the attentional deficits characteristic of neglect patients with damage to the rTPJ region may not be explainable unless the focus of neglect research is widened to include the effects of brain networks whose primary function is not attention.

In terms of social cognition, the alternative accounts we focus on here have emphasized the notion that mechanisms for external attention have been evolutionarily coopted to play a role in social cognition (Decety and Lamm, 2007; Corbetta et al., 2008). In contrast, we hypothesize that mentalizing (i.e., our capacity to represent the internal mental states of conspecifics) was built upon a system for internal attention, e.g., whose original functions were those of interoception and self-regulation. According to our account, this system evolved to be in tension with a system for representing the physical and mechanical properties of inanimate objects, which are built upon systems for external attention, e.g., perception and the manipulation of objects. Our account of mentalizing as coopting mechanisms for internal attention fits best with the anatomy of medial parts of the DMN associated with mentalizing (dMPFC and MP/PC). The evidence from rs-fcMRI and activation studies strongly suggests the AG is part of the same network as these medial regions, however, it's anatomical location is less congruent with a connection to internal attention. Instead, the right AG lies near to a right lateralized system of occipital and temporal regions involved in the sensory processing of socially relevant information (Kanwisher et al., 1997; Peelen, 2004; Pelphrey, 2005). In other words, the posterior TPJ may represent a critical junction box where different types of social information are integrated, namely information that derives from internal attention (medial DMN regions) and external attention (right lateralized regions for social perception). This fits well with the posterior TPJ's more specific functional role in representing the intentions of perceived agents (Saxe and Powell, 2006; Saxe et al., 2006).

This raises an interesting question: might there be an evolutionary reason for the tension between posterior and anterior TPJ regions? While such an account would be speculative, it does seem that there are good reasons for a region with the function of posterior TPJ to have an inhibitory connection with regions involved in visual search, and for its activity to increase when an unexpected stimulus is detected. Outside the laboratory, suddenly appearing unexpected stimuli are often animals or conspecifics, which might pose a survival threat. Attempting to find one more apple is not so important as attending to the danger posed by a predator. In this scenario, there is not only an advantage to breaking the current attentional set, there is also an advantage to expediting the processing of social cues and rapidly generating a model of the agent's intentions. Hence, while there is no obvious feature of laboratory reorienting tasks which calls for the engagement of social processing; this may nonetheless occur because the engagement of social processing upon detection of a salient unexpected stimulus is adaptive as a general rule. Consistent with this speculative account, there is evidence that animate motion captures attention more rapidly than inanimate motion (Pratt et al., 2010). If this account is borne out, then it may be that information is indeed passed from social processing areas in the posterior TPJ to the DAN in order to reorient attention. Our hypothesis is that this information would derive from active anticipation of the likely actions of a perceived agent using ToM. Hence, surprisingly, many of the functions attributed to the rTPJ by the VAN account are consistent with the account offered here. The major difference is that we hypothesize these reorienting functions evolved because of evolutionary pressure for more sophisticated social processing, and our accounts predicts these function will be most profitably investigated using realistic social paradigms.

Distinguishing between these accounts is clearly theoretically significant for our understanding of cortical function. In addition, it has implications for therapeutic approaches. If it is correct that attentional reorienting represents a basic process which is coopted for social cognition, then this would suggest that early intervention by training attention might be an effective treatment for individuals with social deficits, such as individuals with Autism Spectrum Disorders. On the other hand, if our account is correct, then non-social attention training programs are not likely to be effective for improving social function, and may even be detrimental.

## Conclusions

For more than a decade, the theory of the ventral attention system has played a leading role in the interpretation of findings which implicate the rTPJ in attention and social processing. In this paper we propose an alternative account which appeals to the interplay between two distinct regions at the rTPJ which are associated with antagonistic functional networks involved in social and non-social processing. We present empirical evidence which is more consistent with this alternative account than prior accounts, identifying distinct loci and functional connectivity maps associated with target detection, reorienting and ToM. We acknowledge this evidence is limited in scope, relying entirely on meta-analysis and rs-fcMRI. It does not make use of experimental manipulation of the processes under investigation, high-resolution imaging, or analysis of individual participants, all of which we expect to be critical to establishing a definitive account. However, these findings do motivate further consideration of our account, which has significant implications. First, it has the potential to make sense of a large and confusing literature on the role of the rTPJ in attention and social processing. Second, it suggests an alternative view of the evolution of brain function, in particular functions associated with social cognition. Third, our account emphasizes attempts to understand neural activity not just by reference to the immediate demands of the experimental task, but also by reference to constraints which our neural structure places on cognition. Task analysis of attention reorienting paradigms does not suggest any role for social processing. Nonetheless, we suggest that activation patterns associated with these paradigms cannot be fully understood without reference to an inbuilt neural tension between focused attention and social processing.

### Conflict of interest statement

The authors declare that the research was conducted in the absence of any commercial or financial relationships that could be construed as a potential conflict of interest.

## Footnotes

^1^This follows from the fact that meta-analytic investigations are based on information about activation peaks, which are not informative about the spatial extent of activation. Further, variations in individual anatomy and in atlas registration for different studies mean that even conditions with distinct peak loci may not be resolved and appear to overlap. On the other hand, if formal meta-analysis reveals a significant difference in location between conditions, then a secure inference can be made that the conditions have spatially distinct activation profiles, because the location of peaks is informative about the spatial distribution of activation and random variations in anatomy contribute to the error term.

^2^Scholz et al. (2009)'s title might be read as implying the existence of two regions that they demonstrate are functionally distinct. However, their own evidence suggests functional overlap, since their attention reorienting region is modulated by ToM and their ToM region is modulated by attention reorienting. Scholz et al. (2009) do not present a statistical analysis that addresses the issue of whether the regions they identify are functionally overlapping or distinct. This would require demonstrating an interaction with spatial location, where the spatial locations are identified on the basis of independent data. They do present a statistical analysis based on individual subject analysis which supports the claim the conditions are associated with distinct peak activations. This finding is consistent with findings we report, and with the view that there is functional overlap between ToM and reorienting.

^3^While the TPN was aligned with the dorsal attention network in Fox et al's initial papers (Fox et al., 2005, 2006) the spatial characterization of the TPN in those analyses was constrained both by negative correlations with seeds in the DMN and by positive correlations with points generated by studies of visual attention. Later studies have more simply identified areas which are negatively correlated with DMN seeds (Chang and Glover, 2009; Fox et al., 2009; Chai et al., 2012; Jack et al., 2012). These regions overlap both the DAN and FPCN.

^4^A concern the reader may have with this account is that it would appear inefficient for the brain to expend energy increasing activity in a region whose function is unrelated to task demands. However, a large body of work indicates the brain is ‘inefficient’ in this way: DMN activity typically increases when non-social task demands terminate (Raichle and Snyder, 2007). Hence, this concern is not specific to the account we give here.

^5^Notably Scholz et al. (2009) only found a very small area of significant activation associated with attention reorienting in their group analysis, even though they had a relatively large number of participants (*n* = 21). This suggests that their implementation of the attention reorienting paradigm was different from other groups, who have identified more extensive activations.

^6^This represents an important methodological concern, however the reader should note that the negative correlations we report are validated by other methods. First, a number of laboratories have observed anti-correlations using conservative methods that don't employ mean signal regression (Chang and Glover, 2009; Fox et al., 2009; Chai et al., 2012; Jack et al., 2012). Second, Jack et al. (2012) validate anti-correlations derived from resting connectivity by demonstrating that they correspond with task related activations and deactivations seen in both the DMN and TPN. Finally, it is important to note that conservative methods which do not use a global regressor likely underestimate the degree of true anti-correlations, and that findings using a global regressor appear more accurate when compared to independent evidence: The methods of Fox et al. (2005) using global normalization, which we also use here, demonstrate good correspondence with regions that are consistently deactivated during cognitively demanding non-social tasks (Raichle and Snyder, 2007).
